# Comparison of bacterial culture with BioFire® FilmArray® multiplex PCR screening of archived cerebrospinal fluid specimens from children with suspected bacterial meningitis in Nigeria

**DOI:** 10.1186/s12879-023-08645-7

**Published:** 2023-10-02

**Authors:** S. Obaro, F. Hassan-Hanga, N. Medugu, R. Olaosebikan, G. Olanipekun, B. Jibir, S. Gambo, Theresa Ajose, Carissa Duru, B. Ebruke, H. D. Davies

**Affiliations:** 1 Division of Pediatric Infectious Diseases, University of Nebraska Medical Center, Omaha, NE US; 2 International Foundation Against Infectious Diseases in Nigeria (IFAIN), Abuja, Nigeria; 3 Department of Pediatrics, Aminu Kano Teaching Hospital, Bayero University/ Bayero University, Kano, Nigeria; 4 Department of Medical Microbiology and Immunology, Nile University of Nigeria, Abuja, Nigeria; 5 Department of Pharmacology & Experimental Therapeutics, Thomas Jefferson University, Philadelphia, PA US; 6 Hasiya Bayero Pediatric Hospital, Kano, Nigeria; 7 Department of Pediatrics, Murtala Mohammed Specialist Hospital, Kano, Nigeria

**Keywords:** BioFire® FilmArray®, Meningitis, Encephalitis, *H. influenzae*, *N. meningitidis* and *S. pneumoniae*

## Abstract

**Background:**

Diagnosis of bacterial meningitis remains a challenge in most developing countries due to low yield from bacterial culture, widespread use of non-prescription antibiotics, and weak microbiology laboratories. The objective of this study was to compare the yield from standard bacterial culture with the multiplex nested PCR platform, the BioFire® FilmArray® Meningitis/Encephalitis Panel (BioFire ME Panel), for cases with suspected acute bacterial meningitis.

**Methods:**

Following Gram stain and bacterial culture on cerebrospinal fluid (CSF) collected from children aged less than 5 years with a clinical suspicion of acute bacterial meningitis (ABM) as defined by the WHO guidelines, residual CSF specimens were frozen and later tested by BioFire ME Panel.

**Results:**

A total of 400 samples were analyzed. Thirty-two [32/400 (8%)] of the specimens were culture positive, consisting of; three *Salmonella* spp. (2 Typhi and 1 non-typhi), three alpha hemolytic *Streptococcu*s, one *Staphylococcus aureus*, six *Neisseria meningitidis*, seven *Hemophilus influenzae*, 11 *Streptococcus pneumoniae* and 368 were culture negative. Of the 368 culture-negative specimens, the BioFire ME Panel detected at least one bacterial pathogen in 90 (24.5%) samples, consisting of *S. pneumoniae, N. meningitidis* and *H. influenzae*, predominantly. All culture positive specimens for *H. influenzae*, *N. meningitidis* and *S. pneumoniae* also tested positive with the BioFire ME Panel. In addition, 12 specimens had mixed bacterial pathogens identified. For the first time in this setting, we have data on the viral agents associated with meningitis. Single viral agents were detected in 11 (2.8%) samples while co-detections with bacterial agents or other viruses occurred in 23 (5.8%) of the samples.

**Conclusions:**

The BioFire® ME Panel was more sensitive and rapid than culture for detecting bacterial pathogens in CSF. The BioFire® ME Panel also provided for the first time, the diagnosis of viral etiologic agents that are associated with meningoencephalitis in this setting. Institution of PCR diagnostics is recommended as a routine test for suspected cases of ABM to enhance early diagnosis and optimal treatment.

## Introduction

Acute bacterial meningitis (ABM) is the most common infection of the central nervous system and remains a major cause of mortality and morbidity in several countries in sub-Saharan Africa [1–5]. The annual estimate for the burden of ABM is 1.2 million each year worldwide [6–8]. While the incidence in most developed countries have declined significantly due to the wide implementation of pneumococcal and *Haemophilus influenzae* conjugate vaccines, such decline is yet to be accomplished in most developing countries where the bacterial etiologic agents are more diverse and diagnostic services are sparse. Further, the sporadic and unpredictable outbreaks of meningococcal meningitis in Africa also contribute significantly to this burden both in children and adults [2, 9–11].

While the diagnostic work-up typically starts with identification of classic symptoms, such as fever, headache, neck stiffness, these are largely unreliable in young children and therefore fraught with significant risk of missed diagnosis [12]. Even when ABM is associated with sepsis, appropriate treatment for sepsis may be inadequate for the accompanying ABM especially as the agents are generally different [13], hence the need for confirmatory diagnosis by the examination of cerebrospinal fluid (CSF) and identification of etiologic agent is essential to guide optimal management. Conventional methods such as Gram stain and culture have very limited value even when standard microbiology laboratories are readily available [14]. In most developing countries, microbiology diagnostic laboratories are poorly equipped, trained personnel are scarce and the turnaround time for these procedures are often suboptimal for optimizing patient care [14]. Thus, the use of non-culture based, rapid tests are needed in these settings to improve diagnostics and optimize patient management. Multiplex polymerase chain reaction (PCR) is one such approach that can improve the detection of bacterial, viral, and fungal agents in the CSF through the detection of microbial nucleic acids, including from non-viable organisms [15, 16]. Although in recent years, PCR based testing has become more readily available at national reference laboratories in several countries within the meningitis belt of Africa, these focus primarily on the detection of bacterial pathogens and are often restricted to the meningitis season [11, 16]. The aim of this study was to compare the yield from standard bacterial culture with the multiplex nested PCR platform, the BioFire® FilmArray® Meningitis/Encephalitis Panel (BioFire ME Panel). We will also aim to determine the clinical usefulness of multiplex PCR for the simultaneous detection of common pathogens associated with meningitis in archived CSF samples of suspected cases of meningitis in young children who were evaluated for community-acquired bacteremia syndromes in Nigeria in comparison with standard diagnostics. We utilized the US Food and Drug Administration (FDA) cleared multiplex PCR (mPCR) platform, the BioFire® FilmArray® Meningitis/Encephalitis Panel (BioFire ME Panel) (BioFire Diagnostics, LLC, Salt Lake City, UT) which tests for six bacteria (*Escherichia coli* K1, *H. influenzae*, *Listeria monocytogenes*, *Neisseria meningitidis, Streptococcus agalactiae*, and *Streptococcus pneumoniae*) seven viruses (cytomegalovirus [CMV), enterovirus [EV), herpes simplex virus type 1 [HSV-1), herpes simplex virus type 2 [HSV-2), human herpes virus type 6[(HHV-6), human parechovirus [HPeV), and varicella zoster virus [VZ))) and one yeast (*Cryptococcus neoformans/gattii)*. Using this platform, results are generated in about an hour for each sample.

## Materials and methods

### Enrollment sites

The enrollment sites at Federal Capital Territory (FCT) are as previously described [17, 18]. In Kano we enrolled children from Aminu Kano Teaching Hospital, Hasiya Bayero Paediatric Hospital, and Murtala Mohammed Specialist Hospital. While Aminu Kano Teaching Hospital serves as a tertiary referral center, the other 2 facilities provide primary and secondary care services. The combined outpatient attendance for children at these 3 facilities is about 1000 daily. Kano is the capital of Kano state in northwest Nigeria and one of the oldest cities in the country. Although largely urban, Kano is not as cosmopolitan as Abuja but is more densely populated. The entire state is within the meningococcal disease belt. Malaria transmission is seasonal [19], and HIV prevalence among women attending antenatal clinic is 1.3% [20, 21].

### Participant enrollment

Trained study physicians and research assistants were stationed in the emergency units, clinics, and wards of the participating hospitals from where they enrolled participants. Eligible patients were those aged less than five years and presenting with fever of > 3 days duration in addition to prostration, convulsion, refusal to feed, diarrhea, or altered consciousness were eligible. Signed informed consent was obtained from the parent or guardian. A physician administered a detailed questionnaire to obtain information on clinical history and physical examination findings.

#### Clinical specimen collection and initial processing

In children with a clinical suspicion of meningitis, CSF was collected through lumbar puncture in a labelled sterile vial by a medical officer under aseptic conditions and transported to the microbiology laboratory, no longer than 2 h after collection. From specimens with total volume over 2 mL, aliquots of the specimens were aseptically transferred and stored in cryovials at minus 80 °C for future diagnostic testing.

A few drops of a centrifuged portion of the sample were placed on a clean glass slide, air dried, heat fixed, Gram stained and examined for blood cells and bacteria. A loopful of the sample was inoculated onto 5% sheep blood agar, chocolate agar, and MacConkey agar. The 5% sheep blood agar, and chocolate agar plates were incubated in 5% CO_2_ while the MacConkey agar was incubated in ambient air. Incubation for all culture media was at 37˚C for 24 to 48 h. After incubation, bacteria were identified by their colony morphology, phenotypic tests, and limited biochemical testing such as oxidase test, indole test, citrate test and TSI tests were performed. Rapid antigen detection kits, viral or fungal culture facilities were not available and hence, these tests are not performed.

Cerebrospinal fluid specimens (*n* = 400) originally collected from the Community-acquired bacteremic syndromes in Young Nigerian Children (CABSYNC) and Community-acquired Pneumonia and Invasive Bacteria Disease (CAPIBD) projects were submitted for this study. In addition to these we also processed residual samples collected from patients in the northwest zone of the country during meningitis outbreaks.

Samples met inclusion criteria if they had not been centrifuged, had a residual volume of ≥ 200 μl and are not visibly blood stained. All specimens were collected between January 2014—January 2018, stored at minus 80˚C before use and did not undergo any freeze–thaw cycles prior to testing by BioFire® ME Panel.

#### Ethical approval

This study was reviewed and approved by the Federal Capital Territory (FCT) Ethics Committee (approval number FCTA/HHSS/NH/GEN/54/II/128) and the Ethics Review Committee of the Kano State Health Services Management Board (Approval numbers AKTH/MAC/SUB/12A/P-3/VI/972 and NHREC/21/08/2008/AKTH/EC/872).

### BioFire® ME Panel testing

Approximately 200 µl of CSF was subjected to testing with the BioFire® ME Panel according to the manufacturer’s instructions. The closed FilmArray system performs nucleic acid extraction, reverse transcription, and multiplex nested PCR, automatically interprets endpoint melting curve data and provides the result in approximately 1 h. The BioFire® ME Panel tests for genetic targets from *E. coli* K1, *H. influenzae*, *L. monocytogenes*, *N. meningitidis*, *S. agalactiae*, *S. pneumoniae*, CMV, EV, HSV-1, HSV-2, HHV-6, HPeV, VZV and *C. neoformans/gattii.*

Each BioFire® ME panel pouch contains two internal controls; an RNA process control that targets RNA transcript from the yeast *Schizosaccharomyces pombe* (freeze-dried into the pouch and rehydrated upon addition of sample, which was carried through all stages of the test process) and a PCR2 control that detects a DNA target dried into the wells of the array (which ensures that the second stage PCR is successful). A run is considered successful only if the two internal controls are valid.

Upon installation of the equipment at the International Foundation Against Infectious Diseases in Nigeria (IFAIN) Central Laboratory in Abuja, five frozen external controls mixes, covering all 14 targets, were provided for daily testing. FilmArray operators completed a valid external control run on each day of specimen run.

BioFire® ME Panel performance: The procedure for testing the specimens proceeded without any hitches and was uneventful.

### Verification of results from specimens with mixed bacterial agents

The AriaMx Real-Time PCR System is an integrated quantitative PCR system which performs amplification, detection, and analysis within the same equipment. It consists of inbuilt thermal cycler, an optical system with an LED excitation source and comprehensive data analysis program. The instrument holds up to six modules. The scanning optics design is able to deliver optimal separation between the dyes and the samples. The PCR system has a closed-tube detection format that can be used with a range of fluorescence detection chemistries including SYBR Green and EvaGreen dyes as well as flurogenic probe systems including Taqman probes [22, 23].

The 12 samples with mixed bacterial agents were tested using the Direct Real-Time protocol on the AriaMx [24]. This involves making a reaction mixture with 2 μl of neat CSF sample with 12 μl of Quanta Perfecta qPCR ToughMix Low ROX MasterMix, 7.5 μl of PCR grade water, and 1.0 μl each of forward primer, backward primer, and probe, giving a total reaction volume of 25 μl per well. For each of the target bacterial agents, Species-specific primers, and probes (SodC for *N. meningitidis*, hpd *H. influenzae* and LytA for *S. pneumoniae*) were used. Positive DNA controls for each of the target bacteria were included to validate each run, as well as No Template Controls (NTC) as negative controls to check for possible contamination.

At the end of the run, the PCR machine calculates the cycle threshold (Ct) for each gene target by determining the signal strength at which the fluorescence exceeded the threshold limit. The presence or absence of the target gene in each isolate is determined by the Ct value. Specimens with Ct value ≤ 35 were considered positive. Where the specimen yielded a valid Ct value for more than one target gene, the specimen was retested for the target gene involved and was only reported as positive if the repeat test gave the same result as the initial test.

## Results

**Fig. 1:**
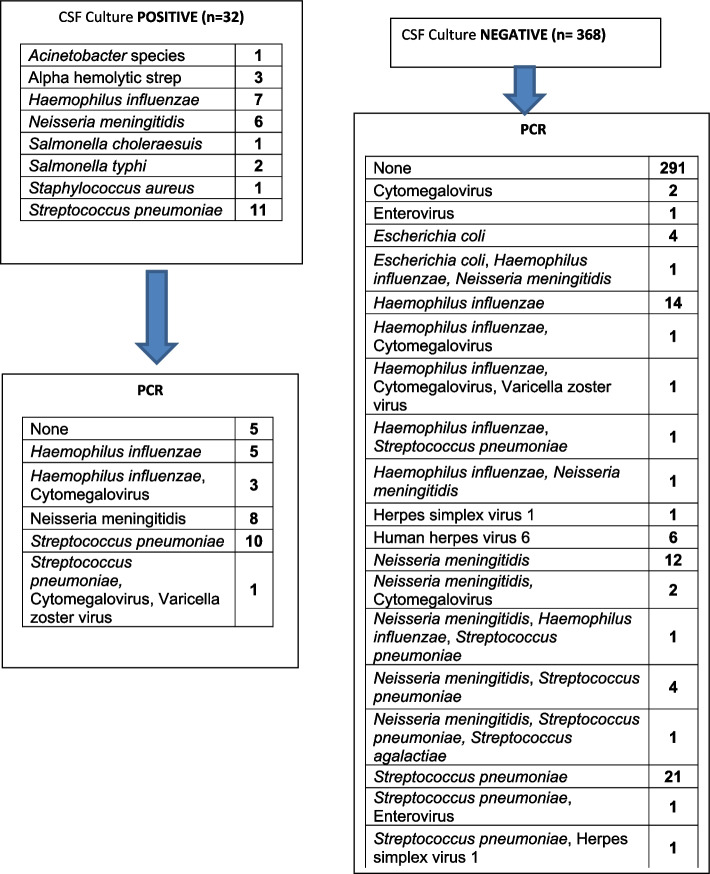
Results summary

Four hundred CSF specimens consisting of 372 (93.0%) from children aged less than 5 years and 27 (6.8%) from an incidental meningococcal disease outbreak in a neighboring state within the northwest zone of Nigeria were included in the study. Over 90% of the samples were collected from Kano, where most children were enrolled from Murtala Mohammed Specialist Hospital (Table 1). Young infants aged less than 2 months constituted 55/400 (13.6%) of the participants (Table 2). Bacterial culture was positive in 32/400 (8%) of the specimens, while 368 specimens were culture negative. The distribution of agents detected from CSF bacterial culture is summarized in Fig. 1.

**Table 1:** Distribution of patients by site of enrollment

*Location*	*Frequency*	*Percent*
Sokoto	10	2.5
Kano-MSH	205	51.2
Kebbi	2	0.5
Kano—Hasiya Bayero	102	25.5
Abuja—Gwagwalada	16	4.0
FMC Keffi	1	0.3
Kano-AKTH	64	16.0
**Total**	**400**	**100.0**

**Table 2:** Age distribution of patients

Age	Frequency	Percent
1–2 months	55	13.8
3 -12 months	119	29.8
13–24 months	68	17.0
25–60 months	130	32.5
> 60 months	27	6.8
Total	399	99.8
Missing	1	0.3
**Total**	**400**	**100.0**

All 400 CSF specimens were tested with the BioFire ME Panel and positive results were obtained from 107/400 (26.75%) specimens. The BioFire ME Panel did not detect any pathogen in 293 (73.25%) samples. The BioFire ME panel detected all bacterial culture positive specimens, except *Acinetobacter, Staphylococcus,* and *Salmonella* which are not on the panel. Single viral agents were detected in 11 (2.7%) samples and a single bacterial agent was detected in 76 (19%) samples. In 10 (2.5%) samples there were mixed bacterial and viral agents detected and 12 (3.0%) samples had mixed bacterial agents detected. Overall, the frequency of pathogens detected was as follows; *S. pneumoniae* 42 (10.5%), *N. meningitidis* 32 (8%), *H. influenzae* 29 (7.3%), *E. coli* 5 (1.3%), *Streptococcus agalactiae* 1 (0.3%). The frequency of viral agents detected was as follows; CMV 10 (2.5%), HHV-6 6 (1.5%), VZV 3 (0.8%), HSV-1 2 (0.5%), and EV 2 (0.5%) (Fig. 1 and Table 3).

**Table 3:** Distribution of pathogens identified by BioFire ME Panel in the cerebrospinal fluid of children with suspected meningitis in Nigeria

**Single Viral Agent**	
*Human Herpes Virus-6*	6
Cytomegalovirus	2
Enterovirus	1
*Herpes simplex virus-1*	1
*Varicella Zoster Virus*	1
***Total***	*11*
**Single Bacterial Agent**	
*S. pneumoniae*	31
*N. meningitidis*	22
*H. influenzae*	19
*E. coli*	4
***Total***	*76*
**Mixed Bacterial and Viral Agents**	
*H. influenzae*, CMV	4
*N. meningitidis*, CMV	2
*H. influenzae*, CMV, VZV	1
*S. pneumoniae*, CMV, VZV	1
*S. pneumoniae*, Enterovirus	1
*S. pneumoniae*, HSV-1	1
***Total***	*10*
**Mixed Bacterial Agents**	
*N. meningitidis, S. pneumoniae*	4
*H. influenzae, S. pneumoniae*	4
*H. influenzae, N. meningitidis*	1
*N. meningitidis, H. influenzae, S. pneumoniae*	1
*E. coli, H. influenzae, N. meningitidis*	1
*N. meningitidis, S. pneumoniae, S. agalactiae*	1
***Total***	12
**Frequency of Detection of Bacterial Agents**	
*S. pneumoniae*	42
*N. meningitidis*	32
*H. influenzae*	29
*E. coli*	5
*S. agalactiae*	1
***Total***	109
**Frequency of Detection of Viral Agents**	
CMV	10
Enterovirus	2
HSV	2
HHV 6	6
VZV	3
***Total***	*23*

**Fig. 2:**
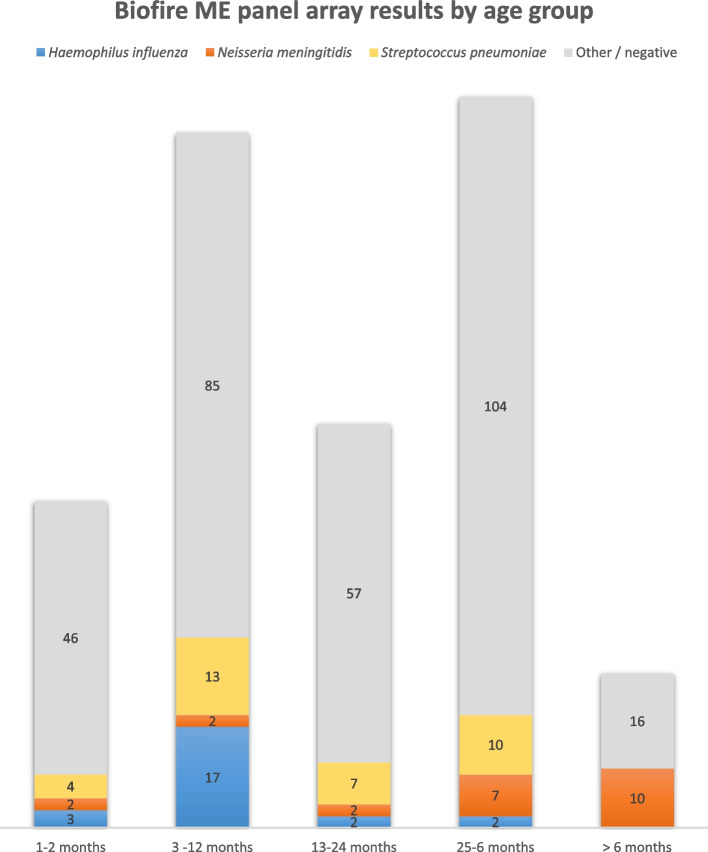
Distribution of etiologic agents by age

Of the 368 bacterial culture negative CSF specimens, 77 (20.9%) were positive with the BioFire ME Panel for at least one bacterial agent. *S. pneumoniae* was the most commonly detected bacterial pathogen by culture (*n* = 11) and by the BioFire ME Panel (*n* = 42). The age distribution of cases identified by BioFire ME Panel reveals a predominant proportion of *H. influenzae* in infants while *N. meningitidis* was more prevalent in older children (Fig. 2). BioFire ME also offers, for the first time from this setting, data on the viral agents associated with meningitis. Single viral agents were detected in 11 (2.8%) samples while co-detections with bacterial agents or other viruses occurred in 23 (5.8%) samples (Table 3).

### Verification of results from specimens with mixed bacterial agents

Those CSF samples which yielded mixed bacterial agents with the BioFire ME Panel were retested using the AriaMx PCR platform as described in materials and methods. There were two samples which did not provide 100% concordance for multiple pathogens detected by BioFire ME Panel when compared to AriaMx PCR. In one, *N. meningitidis* and* S. pneumoniae* were yielded on the BioFire ME Panel but yielded only *N. meningitidis* on the AriaMx PCR platform while in the other, *H. influenzae* and* N. meningitidis* was detected in BioFire ME Panel and *H. influenzae* and *S. pneumoniae* detected in AriaMx PCR. All other multiple pathogens detected by BioFire ME Panel were also detected by AriaMx PCR platform provided the target primers were present in the platform. Hence, they are not analytical false positives as they were detected across multiple platforms. In some cases, the AriaMx PCR platform detected a different pathogen than the culture. This included one *H. influenza* that was identified as alpha-hemolytic Streptococcus, one *N. meningitidis* that was also identified as an alpha-hemolytic Streptococcus by culture, and another *N. meningitidis* that was identified as Acinetobacter species. There were no PCR targets for bacteria isolated in eight of the 32 culture positive specimen (Fig. 1).

We had primarily CSF bacterial culture results on these patients as the source facilities were not resourced to perform CSF chemistry or cell count. The acquisition of clinical outcome data was inconsistent, making it difficult to establish a comprehensive correlation between all laboratory results and patient outcomes. The documentation of clinical outcome data was limited, with most records available only during the initial days of hospitalization. Overall, there were 24 observations which included 7 fatalities (Table 4). These fatalities included two patients with dual infections with *S. pneumoniae* and *N. meningitidis* detected on both PCR panels and in one case detected by the BioFire ME Panel alone without confirmation on the AriaMx.6

**Table 4:** Summary of limited clinical outcome data with CSF culture and PCR results

ID	Outcome	CSF culture	Film Array	AriaMx	age (months)	sex
02–004-1598	D	*S. pneumoniae*	*S. pneumoniae*	*S. pneumoniae, Equivocal N. meningitidis*	24	Male
02–004-1415	D	*H. influenzae*	*H. influenzae*	*H. influenzae*	7	Male
01–021-0590	D	*S. pneumoniae*	*S. pneumoniae*	*S. pneumoniae*	27	Male
02–004-1435	D	*N. meningitidis*	*N. meningitidis*	*N. meningitidis, H. influenzae*	145	Female
02–021-0355	D	No growth	*N. meningitidis, S. pneumoniae*	*N. meningitidis, S. pneumoniae*	48	Male
02–021-0365	D	No growth	*S. pneumoniae*	*S. pneumoniae*	24	Male
03–019-0436	D	No growth	*N. meningitidis*	*N. meningitidis*	30	Male
03–020-0346	R	No growth	None	Not performed	15	Male
03–019-0666	R	*S. pneumoniae*	*S. pneumoniae*	*S. pneumoniae*	6	Male
03–019-0536	R	No growth	*N. meningitidis,* Cytomegalovirus	*N. meningitidis*	103	Male
03–018-0353	R	No growth	None	Not performed	48	Female
03–016-0370	R	No growth	None	Not performed	48	Female
02–021-0358	R	No growth	None	Not performed	30	Male
02–021-0339	R	No growth	None	Not performed	28	Male
02–021-0317	R	No growth	None	Not performed	23	Male
02–020-0572	R	No growth	None	Not performed	59	Female
02–020-0422	R	No growth	*S. pneumoniae*	*S. pneumoniae*	14	Male
02–004-1824	R	*S. pneumoniae*	*S. pneumoniae*	*S. pneumoniae*	9	Female
02–004-1153	R	*H. influenzae*	*H. influenzae*	*H. influenzae*	4	Male
01–021-0691	R	No growth	None	Not performed	6	Male
01–021-0349	R	No growth	None	Not performed	17	Male
01–021-0333	R	No growth	None	Not performed	16	Female
01–021-0297	R	No growth	None	Not performed	25	Male
01–021-0170	R	*S. pneumoniae*	*S. pneumoniae*	*S. pneumoniae*	13	Female

Outcome- Died (D), Recovered (R)

## Discussion

The typical clinical approach for the management of ABM relies on bacterial culture of CSF, which is widely recognized as the gold standard, even though results are often not available until 48 h or more after specimen collection. In practice, most patients are started on empiric, broad spectrum antibiotics, such as a third-generation cephalosporin, but this may not be indicated in all forms of ABM and additional adjunct therapy may be required in specific forms of meningitis, such as steroids in *H. influenzae* type b or *S. pneumoniae*. Thus, delay in definitive etiologic diagnosis of ABM compromises optimal care. Other rapid methods for pathogen detection such as Gram stain have a broad range of sensitivity and are pathogen and population dependent [14]. CSF pleocytosis has very poor specificity in guiding diagnosis of ABM in general, but particularly in young children as counts may be normal despite a diagnosis of ABM [25, 26]. For the detection of viruses, in patients who present with “aseptic meningitis”, molecular detection offers the only accurate diagnostic approach. For detection of cryptococcal meningoencephalitis, lateral flow cryptococcal antigen tests should be the first line test as numerous studies have demonstrated they are the most sensitive, particularly in the early stages of disease and when fungal burden is low. During the past several years the BioFire ME Panel has been extensively evaluated across several developed country settings and has been widely adopted for routine diagnostics for suspected cases of ME [27–30]. The minimal hands-on time for specimen processing, rapid turnaround time and the small CSF volume required for testing multiple pathogens simultaneously makes it particularly attractive for pediatric use.

Our preliminary data from central and northwest Nigeria were generated from archived CSF specimens that were collected from children who presented with severe clinical symptoms such as high fever, irritability, bulgy fontanel, or seizures. In this analysis of archived collection of CSF samples, we utilized, for the first time in this setting a multiplex PCR platform for the simultaneous detection of both viral and bacterial agents. In contrast to reports from most developed country settings, viral agents such as enterovirus was detected infrequently in our patients. Our results also contrast with observations from Ethiopia, which is also within the meningitis belt of Africa and may have had the pioneer experience of using the BioFire ME Panel. Of 218 CSF samples, 21 (10%) were PCR positive; 4% in neonates,14% in children and 18% in adults. Virus was detected in 57% of the PCR positive samples, bacteria in 33% and fungi in 10% [31]. This contrast may in part be due to the fact that our enrollment was at secondary and tertiary facilities where severely ill children are more likely to present and our criteria included children with overt clinical signs of meningitis.

While most countries have now introduced conjugate vaccines against the leading bacterial agents of ABM and have observed significant decline in the incidence of ABM caused by these agents, pneumococcal conjugate vaccine has only recently been introduced to the National Immunization Program in Nigeria and routine immunization coverage in general, remains very low in most parts of northern Nigeria, including Kano [32], the location that contributed the largest number of specimens for this study. In this report, we reveal for the first time the comparative yield from CSF of bacterial culture and PCR from children with suspected ABM in Nigeria. In addition, this also provides the first report of multiplex PCR testing of CSF obtained from Nigerian children for several viruses. The poor performance of bacterial culture, which is the current routine approach to diagnosis of ABM raises significant concerns about the large number of cases of ABM that may be receiving suboptimal care due to missed diagnosis.

The practical interpretation of a culture-negative, PCR positive CSF specimen is likely due to the high prevalence of non-prescription antibiotic use [17]. This is because while the bacterial pathogens are not viable, the genetic material which is the target of PCR can still be detected. This is particularly likely the case in our study because the children had fever for at least three days prior to enrollment and could thus were likely took antibiotics during that time. This thus may have contributed significantly to the culture-negative but PCR-positive results discovered in this study. Other practical possibilities may include patients who present very early or have lower bacterial inoculum in CSF and thus more likely to have full recovery and less complications or are these false positive tests attributable to transient bacteria DNA in the blood stream following translocation from the sinuses or nasopharynx or contamination during specimen sampling [33]. While these remain possibilities, the context in which clinical specimens were obtained from these children with an acute febrile illness and clinical suspicion for meningitis, a diagnosis of meningitis remains the most likely possibility. The retrospective nature of the current study did not permit for the formal exploration of these possibilities.

Molecular techniques are significantly more effective for establishing the etiologic diagnosis of pyogenic meningitis, although they cannot completely replace conventional tests given the need for bacteria isolation and susceptibility testing for the determination of optimal antibiotic choice for clinical management [34]. All the patients in our series were treated empirically with ceftriaxone once a CSF sample was obtained. While this agent is a good choice for most bacterial agents that are associated with meningitis, such as *S. pneumoniae*, *Salmonella* spp. and *N. meningitidis*, obtaining prompt information on the etiologic agent would have modified duration of treatment for cases of *N. meningitidis*, which can be treated optimally with a shorter course than *S. pneumoniae* or *H. influenzae*. In addition, such information would have prompted the initiation of prophylaxis for household contacts for those children with *N. meningitidis* or prompted evaluation for alternative cause of illness or modification of empiric treatment.

The detection of viral agents by PCR clearly has a strong indication for initiating specific treatment for HSV and supportive care for enterovirus. However, detection of CMV and VZV or HHV6 warrant careful clinical correlation, since these are herpes viruses and detection in CSF may be secondary to reactivation rather than primary infection. The clinical relevance of these agents in this setting where co-morbid conditions such as severe malnutrition and HIV are prevalent warrants further investigation.

There are several limitations for this study. We determined the performance of the BioFire ME Panel on archived CSF specimens, relative to bacterial culture alone. We did not have a local diagnostic platform for the detection of viral and fungal agents that are included in the BioFire ME Panel. While our storage conditions for the CSF are deemed optimal, we are unable to guarantee absence of DNA degradation following prolonged specimen storage; although there are reports in the literature of specimens in storage for over three decades been screened for bacterial DNA yielding meaningful results [35, 36].

We detected a high prevalence of mixed bacterial meningitis in our population. The clinical setting where the patients are triaged and specimens collected are often congested and facemasks are not typically worn during these procedures, thus raising the likelihood of contamination at the point of collection. Mixed bacterial infections are frequently reported following cranial trauma, surgical intervention or in nosocomial infections. Nevertheless, there have been several reports of community acquired ABM with mixed bacterial agents in immunocompetent patients [37, 38]. Most frequently reported are infections caused by a combination of common meningeal pathogens such as *N. meningitidis* and *S. pneumoniae* or *N. meningitidis* and *H. influenzae*. Most of the reports in the literature of polymicrobial ABM predates the widespread use of antibiotics, with prevalence ranging from 1–4%, predominantly affecting young children and the most frequent bacteria being *H. influenzae* [39, 40].

In a recent report of the evaluation of CSF samples from patients with pyogenic meningitis by high-throughput sequencing of 16S rDNA, 61.5% of specimens had mixed pathogens [33]. While this report represents one of the earliest uses of multiplex sequencing in evaluating pyogenic meningitis, the results challenge the current norm of associating pyogenic meningitis with single agents that are identifiable only by culture. Where clinical outcome data is available, these polymicrobial ABM have been associated with high case fatality and neurological sequelae [40]. We were unable to completely exclude the possibility of specimen contamination at the point of collection or specimen aliquoting in the laboratory. It is less likely however, that the specimens were contaminated during BioFire ME Panel processing, since the system is self-contained, and the testing pouch is not open at any time after sample/buffer addition. We further minimized the risk of amplicon contamination by ensuring that operators wore a facemask and preparations were performed in a safety hood. Because the specimens mainly constituted a convenience sample that wasn't drawn from a representative population, drawing conclusions about the varying frequencies of infections caused by different pathogens or the notable proportion of bacterial coinfections identified becomes challenging and will not be accurately deduced from this study.

The BioFire ME panel offers a user-friendly and rapid approach for diagnosis but the cost per test will be unaffordable in low- and middle-income country settings. In the meningitis belt of Africa where outbreaks of meningococcal disease occur, these have been frequently associated with concurrent mini outbreaks of *S. pneumoniae* meningitis and in 2016 there was a predominant outbreak caused by *S. pneumoniae* serotype 1 [41]. The scarcity of diagnostic laboratories in this region makes accurate diagnosis a challenge [12, 41] While we have limited outcome data, it is plausible that in this setting, mixed bacterial meningitis may be contributing significantly to adverse outcomes, and these are underestimated due to poor diagnostics [42]. Interpretation of these results would have been significantly enhanced by a full complement of CSF analytics that include cytology and chemistry. These were not consistently available at our clinical sites. We are now planning a prospective study in this setting to evaluate the short- and medium-term impact of polymicrobial meningitis.

In conclusion, we have provided novel data on the etiologic yield for multiple pathogens detected simultaneously from children with clinical suspicion of meningitis from Nigeria, the most populous country within the meningococcal disease belt and in sub-Saharan Africa. The BioFire ME Panel detected several pathogens that were missed by culture. While the BioFire ME Panel cannot replace conventional laboratory studies and the targets can in fact be further customized for agents commonly associated with ABM in this region, the ease and rapidity of performing this test, offers a unique opportunity to improve our understanding of the epidemiology of meningitis and meningoencephalitis in this setting. Future studies integrating unbiased metagenomics pathogen sequencing CSF chemistry and cytology are planned. These studies will provide novel data that will inform the design of customized ME panels for this region. However, to maximize the deployment of such panels, they would need to be affordable with a positive cost–benefit margin in these settings where meningitis is still highly prevalent and such diagnostic devices are most needed.

## Data Availability

All data is available within this manuscript.
